# Thermoelectric current in topological insulator nanowires with impurities

**DOI:** 10.3762/bjnano.9.107

**Published:** 2018-04-12

**Authors:** Sigurdur I Erlingsson, Jens H Bardarson, Andrei Manolescu

**Affiliations:** 1 School of Science and Engineering, Reykjavik University, Menntavegur 1, IS-101 Reykjavik, Iceland; 2 Department of Physics, KTH Royal Institute of Technology, Stockholm, SE-106 91 Sweden

**Keywords:** topological insulators, nanowires, thermoelectric current

## Abstract

In this paper we consider charge current generated by maintaining a temperature difference over a nanowire at zero voltage bias. For topological insulator nanowires in a perpendicular magnetic field the current can change sign as the temperature of one end is increased. Here we study how this thermoelectric current sign reversal depends on the magnetic field and how impurities affect the size of the thermoelectric current. We consider both scalar and magnetic impurities and show that their influence on the current are quite similar, although the magnetic impurities seem to be more effective in reducing the effect. For moderate impurity concentration the sign reversal persists.

## Introduction

It has been known for quite some time now that the efficiency of thermoelectric devices can be increased by reducing the system size. The size reduction can improve electronic transport properties and also reduce the phonon scattering which then leads to increased efficiency [1]. Interestingly, often the materials that show the best thermoelectric properties on the nanoscale can also exhibit topological insulator properties [2], although the connection between the two properties is not always straightforward [3]. Even though few experimental studies exist on thermoelectric properties in topological insulator nanowires (TIN), many studies have reported magnetoresistance oscillations, both in longitudinal and transversal fields for TINs [4–10].

In its simplest form, thermoelectric current is generated when a temperature gradient is maintained across a conducting material. In the hotter end (reservoir) the particles have higher kinetic energy and thus velocity compared to the colder reservoir. This leads to a flow of energy from the hot to cold end of the system. Under normal circumstances this will lead to particles flowing in the same direction as the energy flow. The charge current can of course be positive or negative depending on the charge of the carriers, i.e., whether they are electrons or holes. Recently, it was shown that in systems showing non-monotonic transmission properties the particle current can change sign as a function of the temperature difference [11]. Sign changes of the thermoelectric current are well-known in quantum dots [12–15] when the chemical potential crosses a resonant state. A sign change of the thermoelectric current can be obtained when the temperature gradient is increased, which affects the population of the resonant level in the quantum dot [16–19].

For topological insulator nanowires one can expect reversed, or anomalous, currents measured in tens of nanoamperes [11], well within experimental reach. Also, since the transport is over long systems, it is much simpler to maintain a large temperature difference of tens of kelvins, compared to the case of quantum dots. In this paper we extend our previous work on thermoelectric currents in TIN [11], by including the effects of impurities, both scalar and magnetic ones. The impurities deteriorate the ballistic quantum transport properties, but as long there are still remnants of the quantized levels, the predicted sign reversal of the thermoelectric current remains visible.

## Results and Discussion

### Clean nanowires

When a topological insulator material, such as BiSe, is formed into a nanowire, topological states can appear on its surface. Recently, such wires in a magnetic field have been studied extensively both theoretically [20–24] and experimentally [5–10,25]. When the nanowires are of circular cross section the electrons move on a cylindrical surface with radius *R*. The surface states of the topological insulator are Dirac fermions, described by the Hamiltonian [20–21,26]

[1]
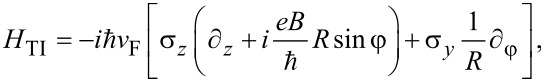


where *v*_F_ is the Fermi velocity, and the spinors satisfy antiperiodic boundary conditions 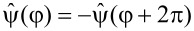 because of a Berry phase [20–21]. We chose the coordinate system such that the magnetic field is along the *x*-axis, **B** = (B,0,0), the vector potential being **A** = (0,0,*By*) = (0,0,*BR*sinφ). For *B* = 0 the angular part of the Hamiltonian has eigenfunctions 
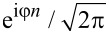
 where *n* are half-integers to fulfill the boundary condition. It is convenient to diagonalize Equation 1 in the angular basis, which are exact eigenstates when *B* = 0.

**Figure 1:**
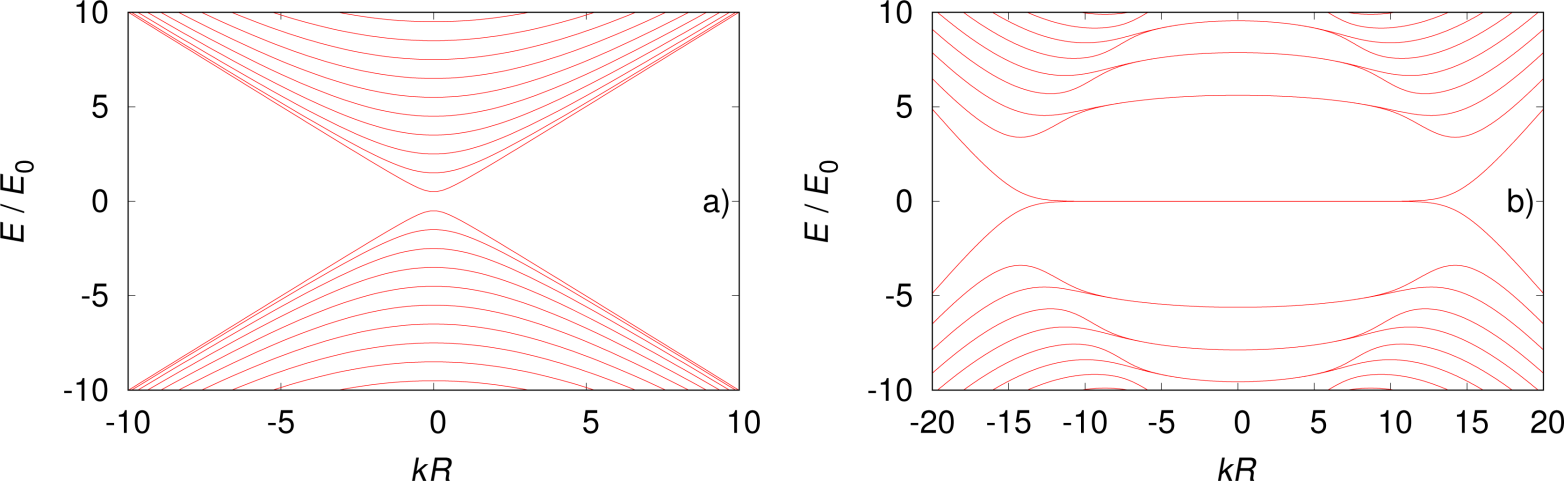
Energy spectra for a) *B* = 0 and b) *B* = 4.0 T. Note that the system is gapped at *B* = 0 but not at *B* = 4.0 T. We used *v*_F_ = 10^5^ m/s and *R* = 50 nm for the current calculations, which gives *E*_0_ = 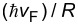 ≈ 1.3 meV.

An example of the energy spectrum is shown in Figure 1 for *B* = 0 (Figure 1a) and for *B* = 4.0 T (Figure 1b). The model parameters are comparable to experimental values [10]. For zero magnetic field the energy spectrum has a gap at *k* = 0 resulting from the antiperiodic boundary conditions [20–21]. For the case of non-zero magnetic fields, precursors of Landau levels around *k* = 0 are seen, both at negative and positive energy. The local minima away from *k* = 0 are precursors of snaking states. Such sates have been studies for quadratic dispersion (Schrödinger) where the Lorentz force always bends the electron trajectory towards the line of vanishing radial component of the magnetic field [27–30]. In fact, this is a classical effect known in the two-dimensional electron gas in inhomogeneous magnetic fields with sign change [31–34]. For Dirac electrons it has been reported in graphene p–n junctions in a homogeneous magnetic field, since in this case the charge carriers change sign [35].

In order to calculate the current in multi-channel one-dimensional systems one needs to calculate the product of the velocity *v_n_*(*E*) and density of states ρ*_n_*(*E*) of a given mode *n* at energy *E* [36]. This product is a constant *v_n_*(*E*)ρ*_n_*(*E*) = 1/*h*, irrespective of the form of ε*_n_*(*k*), which leads to the well-known conductance quantum *e*^2^/*h*. For infinitely long, ballistic systems all channels are perfectly transmitted *T_n_* = 1, so one can simply count the number of propagating modes to obtain the conductance.

If the curvature of the dispersion is negative (here we consider positive energy states) at *k* = 0, then the mode can contribute twice to the conductance since there are two values of *k* that fulfill ε*_n_*(*k*) = *E* and have the same sign of *v_n_*(*E*) (see Figure 1b). The transmission, which in this case is simply the number of propagating modes, can jump up by two unit values and then again fall by one unit value as a function of the energy. As was pointed out recently, the presence of such non-monotonic behavior in the transmission function *T*(*E*) can give rise to anomalous thermoelectric currents [11].

In order clarify the origin of the sign reversal of the thermoelectric current, and how its affected by magnetic field, we will briefly outline how the current is calculated using the Landauer formula. The charge current *I*_c_ is given by

[2]
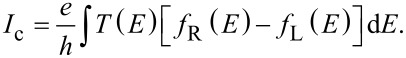


**Figure 2:**
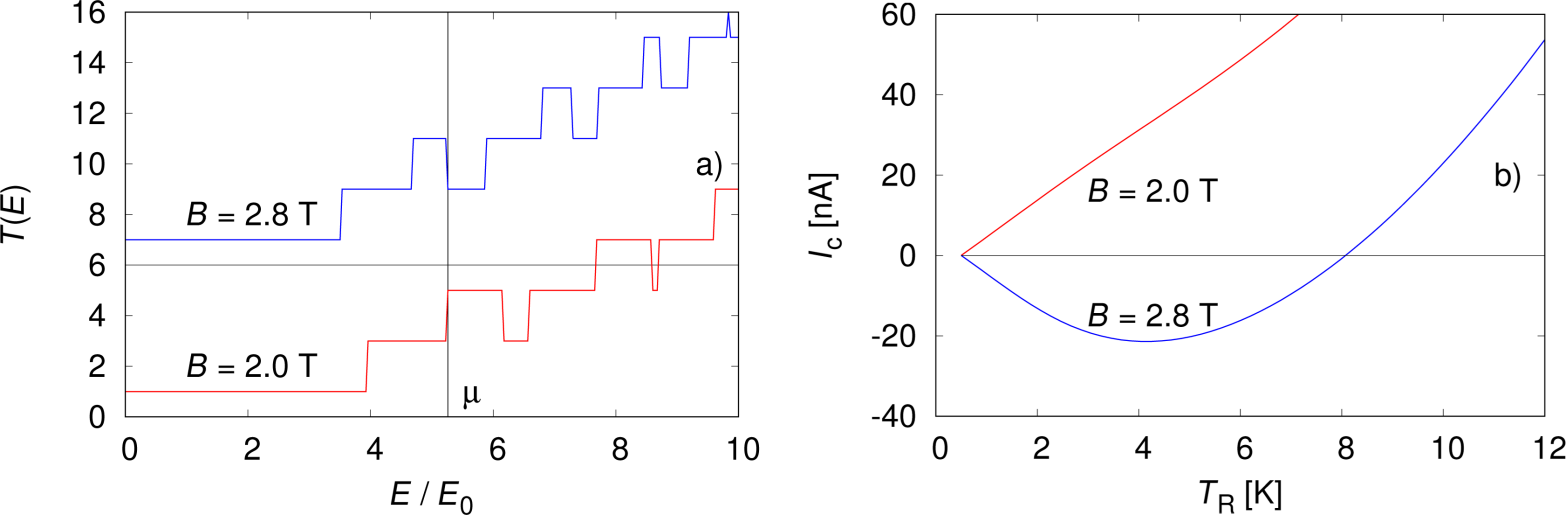
a) Transmission function and b) thermoelectric current for two different magnetic fields. In a), the transmission function *T*(*E*) for *B* = 2.8 T is offset by 6 for clarity. We used *v*_F_ = 10^5^ m/s and *R* = 50 nm for the current calculations, which gives *E*_0_ = 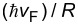 ≈ 1.3 meV.

Here *f*_L/R_(*E*) are the Fermi functions for the left/right reservoir with chemical potentials μ_L/R_ and temperatures *T*_L/R_. We will consider μ_L_ = μ_R_ = μ. If the transmission function *T*(*E*) increases with energy over the integration interval (and the chemical potential is situated somewhere in the interval) the thermoelectric current is positive. This is the normal situation. An anomalous negative current can instead occur if the transmission function decreases with energy. The curve for *B* = 2.0 T in Figure 2a shows the normal situation where the chemical potential is positioned at an upward step at μ = 6.8 meV. The vertical line indicates the position of μ. The resulting charge current is shown in Figure 2b) where the normal situation is evident for *B* = 2.0 T. If the magnetic field is increased to *B* = 2.8 T, the energy spectrum changes (not shown) and so will the transmission function *T*(*E*). Now a downward step occurs at μ, which leads to an anomalous current, as can be seen in Figure 2b. Note that the current sign an be changes by either varying the temperature of the right reservoir or the magnetic field. The anomalous current can be in the range of tens of nanoamperes, which is well within experimental reach.

### Modeling of impurities

The anomalous current introduced above relies on non-monotonic steps in the transmission function. For ballistic nanowires the steps are sharp, but in the presence of impurities the steps will get distorted. In order to simulate transport in a realistic nanowires, we will assume short-range impurities. These are described by

[3]
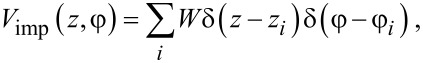


where *W* is the impurity strength. Due to fermion doubling, the Hamiltonian in Equation 1 can not be directly discretized [37]. However, adding a fictitious quadratic term

[4]
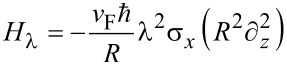


solves the issue of fermion doubling [38]. To fix the value of λ, we will first look at the longitudinal part of Equation 1 in the absence of a magnetic field

[5]
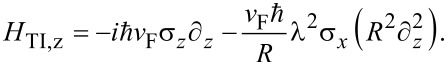


If this Hamiltonian is discretized on a lattice with the lattice parameter *a* the spectrum will be

[6]
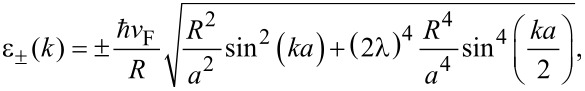


where 
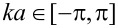
. The value of λ can be set by the condition that the Taylor expansion of (ε_±_(*k*))^2^ contains no quartic term, which maximizes the region showing linear dispersion. This condition is fulfilled when

[7]
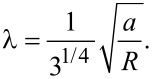


For zero magnetic field we choose the lattice parameter *a* = 0.02 *R*, which ensures that the first ten states calculated via the lattice model with the λ^2^ term deviate by less than 1% from those obtained with the continuum model (Figure 1a). For a non-zero magnetic field we use *a* = 0.01 *R*, because more states contribute to the flat bands at *E* = 0. At this point we are free to use standard discretization schemes and the transmission function in the case when impurities are included is obtained using the recursive Green’s function method [39].

Experiments on normal (not topological) nanowires show a conductance that can be complicated, but reproducible trace for a given nanowire. This means that the measurement can be repeated on the same nanowire and it will give the same conductance trace as long as the sample is kept under unchanged conditions. But a different nanowire would show a different, but reproducible, conductance trace [40]. This motivates us to consider a fixed impurity configuration, i.e., no ensemble average.

**Figure 3:**
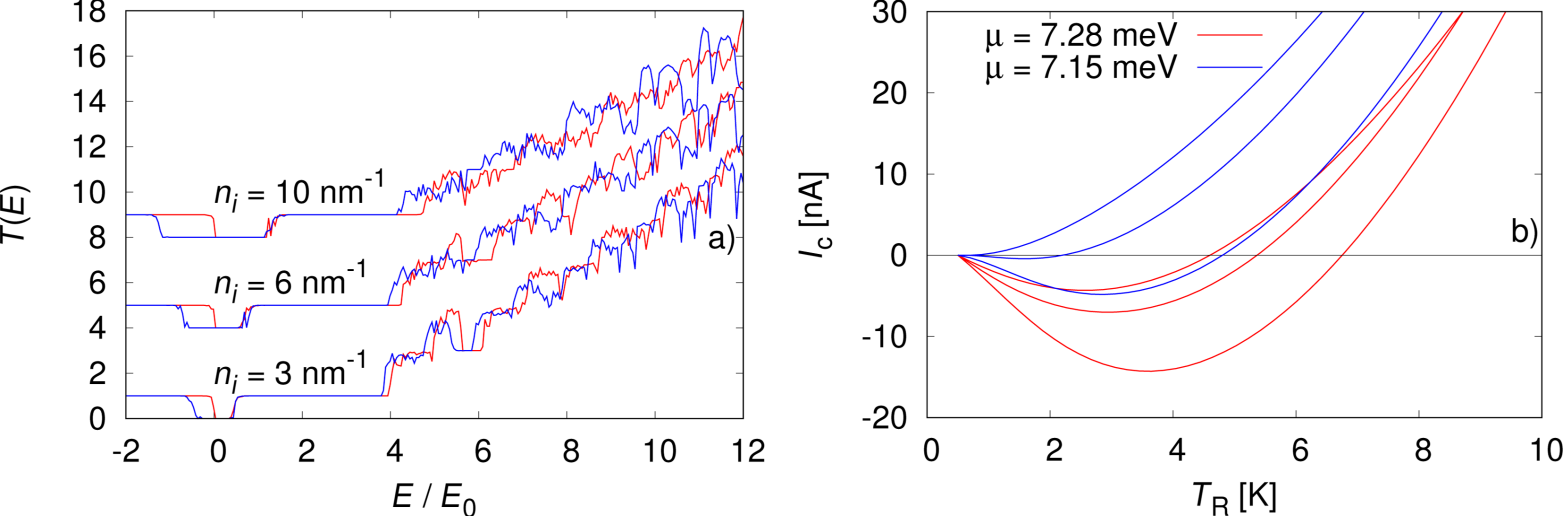
a) Transmission function and b) thermoelectric current calculated in the presence of impurities at *B* = 4.0 T. The nanowire length is *L* = 1000 nm and the impurity densities are *n_i_* =3.0 nm^−1^, 6.0 nm^−1^ and 12 nm^−1^. The red curves are for scalar impurities with chemical potential μ = 7.28 meV and the blue curves are for magnetic impurities with μ = 7.15 meV.

In Figure 3 we show the transmission functions and the thermoelectric currents for a magnetic field of *B* = 4.0 T, for a nanowire of length *L* = 1000 nm. The disorder strength is set to *W* = 4.8 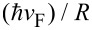 and the density of impurities is varied: *n_i_* = 3.0 nm^−1^, 6.0 nm^−1^ and 12 nm^−1^. For comparison, we consider two types of impurities: scalar impurities described by Equation 3 (red traces), and magnetic impurities described by *V*_imp_σ*_x_* (blue traces).

When the transmission function in Figure 3a in the presence of impurities is studied, a definite trend towards reduced non-monotonic intervals is visible as the density of impurities is increased from 3.0 to 6.0 and 12 nm^−1^. This applies both to scalar (red) and magnetic impurities (blue), even though the magnetic impurities seem to cause a quicker reduction in the transmission peaks. Both scalar and magnetic impurities open up a gap around *E* = 0. This is due to scattering between counter-propagating states on the same side of the nanowire [24]. When looking at the calculated charge current in Figure 3b, the difference between the scalar and magnetic impurities becomes more clear. In both cases the strength and density of impurities is the same but magnetic impurities are substantially more effective in reducing the anomalous current. Note that due to the different impurity configurations between the magnetic and scalar cases we adjusted the chemical potential to μ = 7.15 meV to maximize the anomalous current. The values of *W*_imp_ and *n_i_* used here were chosen such that we could observe an evolution in Figure 3a from resolving the quantized steps to not seeing any. For experiments, this would mean that samples that show some indication of quantized conductance steps should suffice to observe the anomalous current.

In our calculations we neglected the Coulomb interactions between electrons that, in the nonlinear regime of transport, may alter the current, at least in non-topological materials [41–43]. To our knowledge, the present experimental data in TI nanowires can be explained without considering the Coulomb interaction. But, nevertheless, this issue can be an open question for future research.

## Conclusion

We studied the reversal of the thermoelectric current in topological insulator nanowires and how it evolves with changing magnetic fields. Using lattice models we simulated realistic nanowires with both scalar and magnetic impurities. Even though both scalar and magnetic impurities reduce the size of the anomalous current we expect that in quasiballistic samples the effect should be observable. Interestingly, magnetic impurities seem to be more effective than scalar impurities when it comes to reducing the anomalous thermoelectric current. For hollow nanowires described by the Schrödinger equation the backscattering is the same for magnetic and scalar impurities, in the absence of spin–orbit interactions. This is in contrast to the TI nanowires studies here, which are more susceptible to scattering by magnetic impurities due to spin–momentum locking of the surface states [23].

## References

[1] Caballero-Calero O, Martín-González M (2016). Scr Mater.

[2] Pennelli G (2014). Beilstein J Nanotechnol.

[3] Gooth J, Gluschke J G, Zierold R, Leijnse M, Linke H, Nielsch K (2015). Semicond Sci Technol.

[4] Bäßler S, Hamdou B, Sergelius P, Michel A-K, Zierold R, Reith H, Gooth J, Nielsch K (2015). Appl Phys Lett.

[5] Peng H, Lai K, Kong D, Meister S, Chen Y, Qi X-L, Zhang S-C, Shen Z-X, Cui Y (2010). Nat Mater.

[6] Xiu F, He L, Wang Y, Cheng L, Chang L-T, Lang M, Huang G, Kou X, Zhou Y, Jiang X (2011). Nat Nanotechnol.

[7] Dufouleur J, Veyrat L, Teichgräber A, Neuhaus S, Nowka C, Hampel S, Cayssol J, Schumann J, Eichler B, Schmidt O G (2013). Phys Rev Lett.

[8] Cho S, Dellabetta B, Zhong R, Schneeloch J, Liu T, Gu G, Gilbert M J, Mason N (2015). Nat Commun.

[9] Jauregui L A, Pettes M T, Rokhinson L P, Shi L, Chen Y P (2016). Nat Nanotechnol.

[10] Dufouleur J, Veyrat L, Dassonneville B, Xypakis E, Bardarson J H, Nowka C, Hampel S, Schumann J, Eichler B, Schmidt O G (2017). Sci Rep.

[11] Erlingsson S I, Manolescu A, Nemnes G A, Bardarson J H, Sanchez D (2017). Phys Rev Lett.

[12] Beenakker C W J, Staring A A M (1992). Phys Rev B.

[13] Staring A A M, Molenkamp L W, Alphenaar B W, van Houten H, Buyk O J A, Mabesoone M A A, Beenakker C W J, Foxon C T (1993). Europhys Lett.

[14] Dzurak A S, Smith C G, Pepper M, Ritchie D A, Frost J E F, Jones G A C, Hasko D G (1993). Solid State Commun.

[15] Svensson S F, Persson A I, Hoffmann E A, Nakpathomkun N, Nilsson H A, Xu H Q, Samuelson L, Linke H (2012). New J Phys.

[16] Svensson S F, Hoffmann E A, Nakpathomkun N, Wu P M, Xu H Q, Nilsson H A, Sánchez D, Kashcheyevs V, Linke H (2013). New J Phys.

[17] Sierra M A, Sánchez D (2014). Phys Rev B.

[18] Stanciu A E, Nemnes G A, Manolescu A (2015). Rom J Phys.

[19] Zimbovskaya N A (2015). J Chem Phys.

[20] Bardarson J H, Brouwer P W, Moore J E (2010). Phys Rev Lett.

[21] Zhang Y, Vishwanath A (2010). Phys Rev Lett.

[22] Zhang Y-Y, Wang X-R, Xie X C (2012). J Phys: Condens Matter.

[23] Ilan R, de Juan F, Moore J E (2015). Phys Rev Lett.

[24] Xypakis E, Bardarson J H (2017). Phys Rev B.

[25] Arango Y C, Huang L, Chen C, Avila J, Asensio M C, Grützmacher D, Lüth H, Lu J G, Schäpers T (2016). Sci Rep.

[26] Bardarson J H, Moore J E (2013). Rep Prog Phys.

[27] Tserkovnyak Y, Halperin B I (2006). Phys Rev B.

[28] Ferrari G, Goldoni G, Bertoni A, Cuoghi G, Molinari E (2009). Nano Lett.

[29] Manolescu A, Rosdahl T O, Erlingsson S I, Serra L, Gudmundsson V (2013). Eur Phys J B.

[30] Chang C-H, Ortix C (2017). Int J Mod Phys B.

[31] Müller J E (1992). Phys Rev Lett.

[32] Ibrahim I S, Peeters F M (1995). Phys Rev B.

[33] Ye P D, Weiss D, Gerhardts R R, Seeger M, von Klitzing K, Eberl K, Nickel H (1995). Phys Rev Lett.

[34] Zwerschke S D M, Manolescu A, Gerhardts R R (1999). Phys Rev B.

[35] Rickhaus P, Makk P, Liu M-H, Tóvári E, Weiss M, Murand R, Richter K, Schönenberger C (2015). Nat Commun.

[36] Nazarov Y V, Rieth M, Schommers W (2005). Quantum transport and circuit theory. Handbook of Theoretical and Computational Nanotechnology.

[37] Stacey R (1982). Phys Rev D.

[38] Masum Habib K, Sajjad R N, Ghosh A W (2016). Appl Phys Lett.

[39] Ferry D K, Goodnick S M (1997). Transport in Nanostructures.

[40] Wu P M, Gooth J, Zianni X, Svensson S F, Gluschke J G, Dick K A, Thelander C, Nielsch K, Linke H (2013). Nano Lett.

[41] Sánchez D, López R (2016). C R Phys.

[42] Sierra M A, Saiz-Bretín M, Domínguez-Adame F, Sánchez D (2016). Phys Rev B.

[43] Torfason K, Manolescu A, Erlingsson S I, Gudmundsson V (2013). Phys E (Amsterdam, Neth).

